# Active Stabilization of Interventional Tasks Utilizing a Magnetically Manipulated Endoscope

**DOI:** 10.3389/frobt.2022.854081

**Published:** 2022-04-14

**Authors:** Lavinia Barducci, Bruno Scaglioni, James Martin, Keith L. Obstein, Pietro Valdastri

**Affiliations:** ^1^ STORM Lab United Kingdom, Institute of Robotics, Autonomous Systems and Sensing, School of Electronic and Electrical Engineering, University of Leeds, Leeds, United Kingdom; ^2^ STORM Lab United States, Vanderbilt University Medical Center, Nashville, TN, United States

**Keywords:** medical device, magnetic robots control, endoscopes, robot control, autonomous systems

## Abstract

Magnetically actuated robots have become increasingly popular in medical endoscopy over the past decade. Despite the significant improvements in autonomy and control methods, progress within the field of medical magnetic endoscopes has mainly been in the domain of enhanced navigation. Interventional tasks such as biopsy, polyp removal, and clip placement are a major procedural component of endoscopy. Little advancement has been done in this area due to the problem of adequately controlling and stabilizing magnetically actuated endoscopes for interventional tasks. In the present paper we discuss a novel *model-based* Linear Parameter Varying (LPV) control approach to provide stability during interventional maneuvers. This method linearizes the non-linear dynamic interaction between the external actuation system and the endoscope in a set of equilibria, associated to different distances between the magnetic source and the endoscope, and computes different controllers for each equilibrium. This approach provides the global stability of the overall system and robustness against external disturbances. The performance of the LPV approach is compared to an intelligent teleoperation control method (based on a Proportional Integral Derivative (PID) controller), on the Magnetic Flexible Endoscope (MFE) platform. Four biopsies in different regions of the colon and at two different system equilibria are performed. Both controllers are asked to stabilize the endoscope in the presence of external disturbances (i.e. the introduction of the biopsy forceps through the working channel of the endoscope). The experiments, performed in a benchtop colon simulator, show a maximum reduction of the mean orientation error of the endoscope of 45.8*%* with the LPV control compared to the PID controller.

## 1 Introduction

Colorectal Cancer (CRC) is the third most common cancerous disease worldwide (Joseph et al., 2016; Sung et al., 2021); therefore, prevention and early diagnosis of CRC are crucial. Although Flexible Endoscopes (FEs) have been at the forefront in detection and treatment of CRC (Comas et al., 2016), their main disadvantages are patient discomfort and complexity of use, both associated to the stiffness of the endoscope shaft (Spier et al., 2010; Larsen et al., 2020). This leads to limitations in their ability to diagnose and treat CRC (Valdastri et al., 2012).

The demand for new, less invasive and more sophisticated technologies in the prevention of CRC has increased significantly in the last decades (Slawinski et al., 2015b; Barducci et al., 2020). Minimally invasive technologies (i.e., virtual endoscopy, Wireless Capsule Endoscopes (WCEs)) have become commercially available (Brambs and Juchems, 2003; Valdastri et al., 2012; Kumar et al., 2017). Albeit their encouraging results, their main limitation lies in the inability to perform interventional tasks such as biopsy and polyps removal (Valdastri et al., 2012; Obstein and Valdastri, 2013).

In the last decade, new advanced flexible endoscopes (or soft-tethered capsules), have been investigated to overcome WCEs limitations. The presence of a soft-tether enables the use of the endoscope as diagnostic and therapeutic instrument (Slawinski et al., 2015a) and, thus, permits the use of advanced flexible endoscopes as a complete replacement for conventional endoscopes. Moreover, the soft-tether reduces the tissue stretching and, consequently, the discomfort for the patient.

In order to control and navigate an advanced flexible endoscope, an external or internal actuation mechanism is required; this has led to investigating magnetically actuated endoscopes (Sliker et al., 2015). These have major advantages of potential miniaturisation, avoiding complex and bulky internal actuation and achieving minimal invasiveness, leading to a reduction of patient discomfort and potentially decreasing post-operative recovery (Obstein and Valdastri, 2013).

The control of magnetic endoscopes for colonoscopy has mainly been focused on navigation, with the aim of increasing the level of autonomy and reducing the operator burden (Pittiglio et al., 2019; Martin et al., 2020); however, interventional procedures such as biopsy, polyp removal and clip placement, common in clinical colonoscopy (Valdastri et al., 2008; Norton et al., 2019; Zhang et al., 2021), did not receive the same interest in the robotic community. Among interventional tasks, the most common is biopsy. In this procedure, an instrument is introduced through the operative channel to the endoscope tip, where a sample of tissue is collected thorough a pair of forceps.

For tethered devices, performing a biopsy involves passing a flexible instrument through the working channel, aligning the forceps to the target and grasping a portion of the tissue. Biopsies can be categorised in random sampling and targeted procedures (Peixoto et al., 2015). Random biopsies are performed in multiple colon regions and at specific intervals, while targeted biopsies are performed on suspected lesions. Herein, we focus on targeted biopsies, which, having a precise positional target, requires greater accuracy in endoscope positioning and disturbance rejection.

Conventionally, an assistant inserts the forceps while the clinician stabilizes the endoscope. Maintaining a correct alignment between the biopsy forceps and the target is challenging because the endoscope stability is affected by the disturbance caused by the instrument insertion. Autonomously controlling the endoscope orientation during the biopsy procedure would enable the physician to perform a biopsy without the support of an assistant, accelerating the process, improving the accuracy and decreasing the burden on the team.

Few papers focus on robotic biospy: (Simi et al., 2013; Yim et al., 2014; Hoang et al., 2020) propose different approaches in wireless devices, while (Martin et al., 2021; Zhang et al., 2021) developed semi-autonomous routines for performing biopsies, (Zhang et al., 2021) focused on a crawler robot and (Martin et al., 2021) on a magnetic endoscope. However, no previous work could be found in literature on the topic of active stabilization of tethered robotic endoscopes. The task of active stabilization has been addressed only in the content of wireless capsules (Warren et al., 2012; Sodergren et al., 2014; Lee et al., 2017) and in the context of robotic surgery, by means of image-based algorithms that rely on the horizon stabilization principle. In this paper we propose an approach to control magnetic endoscopes during targeted biopsies based on localization sensors, capable of autonomously stabilizing the endoscope in the presence of external disturbances, with a focus on the disturbances created by the instrument insertion.

Magnetic manipulation is based on the interaction between magnetic fields, which is non-linear and varies significantly with the inter-magnetic distance (i.e., the distance between the actuating source of magnetic field and the driven magnet). To provide satisfactory results with a task such as stabilization at different distances, classical techniques like PID and linear controller synthesis would not provide sufficient performance: the PID controller, which needs to be tuned manually, is difficult to adapt to varying parameters and cannot manage multiple controlled variables simultaneously, on-the-other-hand, a classical linear regulator would not guarantee the stability of the overall system when far from the chosen linearization point.

Therefore, the Linear Parameter Varying (LPV) control strategy (Lee, 1997) is considered here. This control strategy involves obtaining linearized dynamic models for the non-linear system at different operating points, described by parameters which slowly vary with respect to the dynamics of the system; then, a Linear Time-Invariant (LTI) control law is designed to satisfy local performance objectives for each operating point. Tuning a controller for each point of equilibrium permits to optimize the closed-loop system in each condition and consequently achieve a general robust stability for the original non-linear system. As the parameters change, the control action is discontinuously switched between the various controllers. Under the condition of slowly changing parameters, the nonlinear system is guaranteed to be globally stable (Apkarian and Noll, 2006).

**FIGURE 1:**
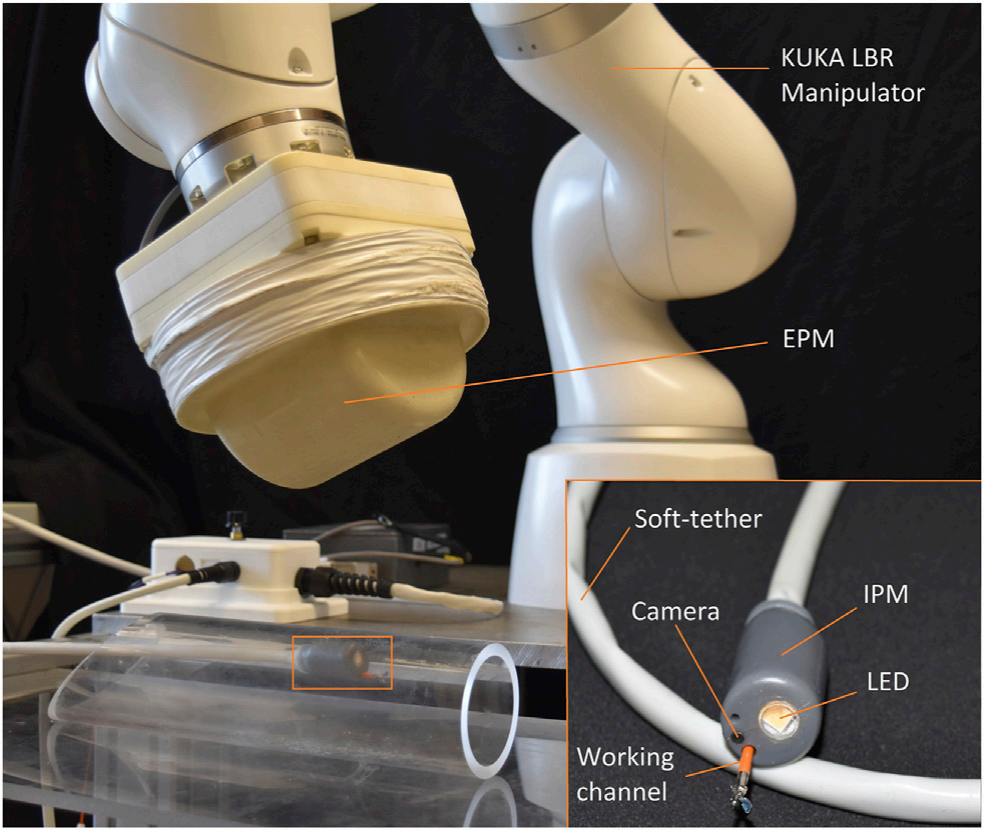
Overview of the MFE system. The magnetic endoscope (bottom right) contains a camera, LED, and a working channel. A KUKA LBR Med robotic arm actuates the MFE via manipulating an external permanent magnet mounted to its end-effector.

The technique is experimentally validated on the Magnetic Flexible Endoscope (MFE) (Martin et al., 2020), an innovative magnetic colonoscope shown in Figure 1, with the aim of stabilizing the magnetic endoscope to perform biopsies. A single soft-tethered endoscope equipped with an Internal Permanent Magnet (IPM) is actuated by means of a robotically manipulated External Permanent Magnet (EPM). Herein, a localization algorithm is embedded to localize the endoscope with respect to the base of the robotic manipulator (Taddese et al., 2018).

This paper is organized as follows: in Section 2 we provide the theoretical formulation of the LPV method. Section 3 presents the experimental results and in Section 4 we discuss the results obtained and further developments of this approach.

## 2 Materials and Methods

**FIGURE 2:**
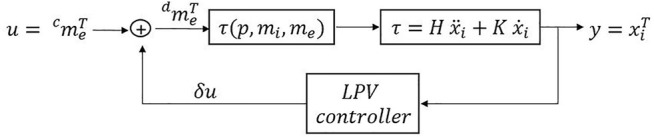
Control scheme. *δu* is the linearized input computed with the LPV controller and meTc and meTd are the current and desired EPM magnetic moment, respectively.

The dynamics of the endoscope is described as a family of parametrized linear systems as in Eq. (1), where *ρ* (*t*) is the parameter, *A*, *B*, *C*, *D* are the matrices describing the system dynamics, *x* (*t*) is the system state, *u* (*t*) is the input and *y* (*t*) is the output. The separation distance between the EPM and the endoscope plays a relevant role in the ability of the EPM to impart a meaningful torque on the IPM and stabilize the endoscope, therefore, it is chosen as time-varying parameter. The closed loop LPV system is described in Figure 2 where the input *u* is the EPM magnetic moment and the controlled variable *y* is the IPM orientation.

$$\left\{\begin{array}{cc} \dot{x}\left(t\right) & =A\left(\rho \left(t\right)\right)x\left(t\right)+B\left(\rho \left(t\right)\right)u\left(t\right)\\ y\left(t\right) & =C\left(\rho \left(t\right)\right)x\left(t\right)+D\left(\rho \left(t\right)\right)u\left(t\right) \end{array}\right.$$ (1)

The dynamics of the IPM (and therefore of the endoscope) is subjected to magnetic interaction, approximated by the dipole-dipole magnetic model which assumes point-shaped sources and correctly approximates the real fields at a distance from the sources equal at least to the diameter of the magnets. In this context, the focus is on the magnetic torque exerted by the EPM on the IPM, which can be written as a vector $\tau_{m}\left(p,x_{i},x_{e}\right)\in R^{3}$ :

$$\tau_{m}\left(p,x_{i},x_{e}\right)=\frac{M}{\|p{\|}^{3}}{\hat{m}}_{I}\times D{\hat{m}}_{E}$$ (2)

where $x_{i}\in R^{3}$ is the IPM orientation, $x_{e}\in R^{3}$ is the orientation of the EPM and *p* = *p*_*i*_−*p*_*e*_ is the relative distance between the IPM and EPM, all expressed in the world reference frame. $M=\frac{\mu_{0}\|m_{I}\|\;\|m_{E}\|}{4\pi }$ with $m_{I}=\|m_{I}\|{\hat{m}}_{I}$ and $m_{E}=\|m_{E}\|{\hat{m}}_{E}$ are the magnetic moments of IPM and EPM, respectively; *μ*_0_ is the magnetic permeability of vacuum, $\hat{p}=\frac{p}{\left\|p\right\|}$ , $Z=I-5\hat{p}{\hat{p}}^{T}$ and $D=3\hat{p}{\hat{p}}^{T}-I$ . Herein $I\in R^{3\times 3}$ is referred to as the *identity matrix* and ‖ ⋅‖ is the *Euclidean norm*.

Consider the rotational term of the nominal dynamics of the IPM

$$H\left(x_{i}\right){\ddot{x}}_{i}+K\left(x_{i},{\dot{x}}_{i}\right){\dot{x}}_{i}=\tau_{m}\left(p,x_{i},x_{e}\right),$$ (3)

$H\left(x_{i}\right),\;K\left(x_{i},{\dot{x}}_{i}\right)$ are referred to as *inertia* and *Coriolis matrix* (Siciliano et al., 2008) of the IPM, respectively.

The goal is to find *x*_*e*_ such that *x*_*i*_ approaches a desired value *x*_*d*_, even in the presence of external disturbances (i.e., respiration of the patient or insertion of the biopsy forceps inside the instrument channel).

The Inertia matrix, referred to the IPM frame, is inferred as the Inertial tensor of a cylinder with mass *m* = 0.023 kg, radius *r* = 0.01 m, and height *h* = 0.035 m.

$$I=\left[\begin{array}{ccc} \frac{1}{12}\left(3mr^{2}+mh^{2}\right) & 0 & 0\\ 0 & \frac{1}{12}\left(3mr^{2}+mh^{2}\right) & 0\\ 0 & 0 & \frac{1}{2}mr^{2} \end{array}\right]$$ (4)

The Inertia matrix is determined w.r.t. the world frame as $H=J^{T}IJ$ where *J* is defined as the Jacobian that links the angular velocity to the derivative of the angular orientation computed in the local frame.

The Coriolis matrix, *K* (*x*) is derived from the Christoffel symbols, hence, each component can be written as:

$$c_{ij}=\frac{1}{2}\left[\frac{\partial b_{ij}}{\partial x_{k}}+\frac{\partial b_{ik}}{\partial x_{j}}-\frac{\partial b_{jk}}{\partial x_{i}}\right]$$ (5)

with the addition of a damping factor to the diagonal of the Coriolis matrix which takes into account the damping provided by the tissues interaction.

Therefore, considering the vector of the state variable as $x={\left[x_{i},{\dot{x}}_{i}\right]}^{T}={\left[x_{1},x_{2}\right]}^{T}$ , the overall system, showed in Figure 2, is modelled by:

$$\left\{\begin{array}{cc} {\dot{x}}_{1} & =x_{2}\\ {\dot{x}}_{2} & =H^{-1}\left(\tau_{m}-K{\dot{x}}_{1}\right)=H^{-1}\left(\tau_{m}-Kx_{2}\right) \end{array}\right.$$ (6)

Subsequently, the system is linearized with respect to the state variable *x* and the input $u=m_{e}^{T}=R_{e}^{g}m_{e}^{EPM}$ , the direction of the magnetic moment of the EPM, where $R_{e}^{g}$ is the rotation matrix of the EPM in world frame and $m_{e}^{EPM}$ is the direction of the EPM magnetic moment in EPM frame. Equation (7) shows the linearized matrices of the system.

$$\begin{array}{c} A=\frac{\partial \dot{x}}{\partial x}=\left[\begin{array}{cc} 0 & I\\ 0 & \frac{-K}{H} \end{array}\right]\in R^{6\times 6},\;B=\frac{\partial \dot{x}}{\partial u}\in R^{6\times 3}\\ C=\left[\begin{array}{cccccc} 1 & 0 & 0 & 0 & 0 & 0\\ 0 & 0 & 0 & 0 & 0 & 0\\ 0 & 0 & 1 & 0 & 0 & 0 \end{array}\right],\;D=0_{3\times 3} \end{array}$$ (7)

The system is linearized around more than one operating point with the aim of finding a controller corresponding to each equilibrium. No a priori information on the parameter is required other that its range of variation, which is assumed to vary with limited velocity. This assumption does not hinder the design of the control and guarantees the stability of the overall system (Shamma and Athans, 1990; Apkarian and Noll, 2006). The relative orientation of the two permanent magnets is maintained constant and only the inter-magnetic distance on the z axis, expressed in world frame, is considered as a Degree of Freedom (DOF) of the system. This is a reasonable assumption, considering that it is always possible to bring the EPM on top of the endoscope before starting the procedure. The system is linearized in these equilibria:

• *m*_*e*_ = (0, −1, 0)
• *x*_1_ = (−*π*/2, 0, −*π*/2)
• *x*_2_ = (0, 0, 0)
• *p* = (0, 0, *ρ*)

As result, an array of systems, linearized in a different value of *ρ*, is obtained. The extremes of the parameter range are chosen as follows: the minimum value as the safest minimum distance of the EPM from the abdominal wall, the maximum as the distance at which the magnetic torque exerted on the MFE is still appreciable. At the minimum relative distance, the magnetic torque and, thus, the steerability of the endoscope are high; when the relative distance is at its maximum the magnetic torque decreases and in order to maintain the same performances, a controller that provides more energy is required. Therefore, employing different controllers at different points of equilibrium is essential.

**FIGURE 3:**
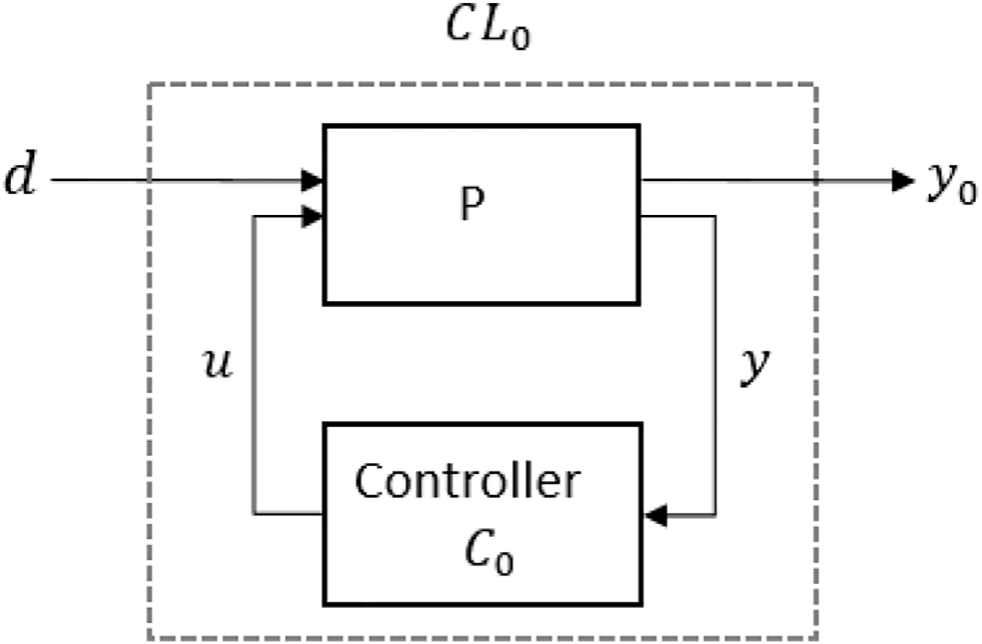
Control scheme represented as LFT system.

To simplify the control system synthesis, the array of systems is expressed in the Standard form model, LPV Linear-Fractional transformation (LFT), as in Figure 3. The LFT form enables to highlight the transfer functions between the disturbances and the system output and consequently synthesize a controller that modifies the effect of disturbances on the output. Here, *d* is referred to a piece-wise constant disturbance that needs to be attenuated and *y*_0_, the output, has to be controlled. *CL*_0_ is the closed-loop system computed given the parametrized LTI systems (defined as *P*) and the controllers *C*_0_ is tuned with a non-smooth optimization algorithm (Apkarian, 2013; Apkarian et al., 2015), available in the Matlab Control System Toolbox. The controllers gain-scheduled on the rho values are matrices 3 × 3, which multiplied to the output of the system (the MFE orientation), compute the desired input (EPM orientation) to reduce the orientation error of the endoscope.

In *y*_0_, the orientation (pitch and yaw) of the IPM is considered. Due to the axial symmetry of the EPM (Taddese et al., 2016), the roll angle is not controllable and, thus, is not considered. As a consequence, the new matrices *B* and *C* are computed as:

$$B_{\mathrm{new}}=\left[\begin{array}{ccc} 1 & 0 & 0\\ 0 & 0 & 0\\ 0 & 0 & 1 \end{array}|B\right]C_{\mathrm{new}}=\left[\begin{array}{c} \begin{array}{cccccc} 1 & 0 & 0 & 0 & 0 & 0\\ 0 & 0 & 0 & 0 & 0 & 0\\ 0 & 0 & 1 & 0 & 0 & 0 \end{array}\\ ―\\ \begin{array}{cccccc} 1 & 0 & 0 & 0 & 0 & 0\\ 0 & 0 & 0 & 0 & 0 & 0\\ 0 & 0 & 1 & 0 & 0 & 0 \end{array} \end{array}\right]$$ (8)

All the conditions for the stabilization of the system and the attenuation of the disturbance *d* are defined appropriately. The disturbance, herein, is defined as a *piece-wise constant* disturbance. This means that velocity and acceleration of the disturbance can be neglected. The assumption made does not interfere nor limit the design of the controller. Considering the type of disturbance tackled in this work, associated with instrument insertion, this assumption is reasonable. In fact, considering a defined amount of time, the instrument insertion can be described as a locally constant disturbance.

**FIGURE 4:**
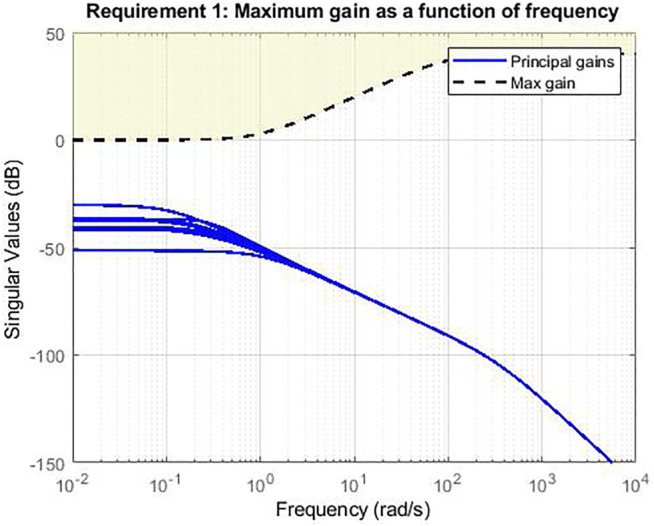
Magnitude Bode diagram of the transfer functions of the MIMO system. Simulation in Matlab with Systune.

The non-smooth optimization algorithm (Apkarian et al., 2015), implemented in the MATLAB-based tool SYSTUNE, is used to find a parametrized controller capable of achieving the required performances on the whole range of parameters. This means maintaining the response between the disturbance and the output of the system under a certain threshold specified by the following high-pass filter transfer function $g=100\frac{\left(s+1\right)}{\left(s+100\right)}$ , which ensures the attenuation of all low frequency signals. The response of the closed loop system is shown in Figure 4. The blue lines represent the transfer functions of the closed loop Multiple-Input Multiple-Output (MIMO) system between the disturbance and the IPM orientation (Euler angles) of the endoscope. For each value of the parameter, considered herein as the inter-magnetic distance, different behaviours are shown in the diagram. It is worth noting that the Bode diagram of the input-output channels of the closed loop system is below the high-pass filter threshold and, thus, the overall non-linear system results as stabilized.

## 3 Experimental Validation and Results

**FIGURE 5:**
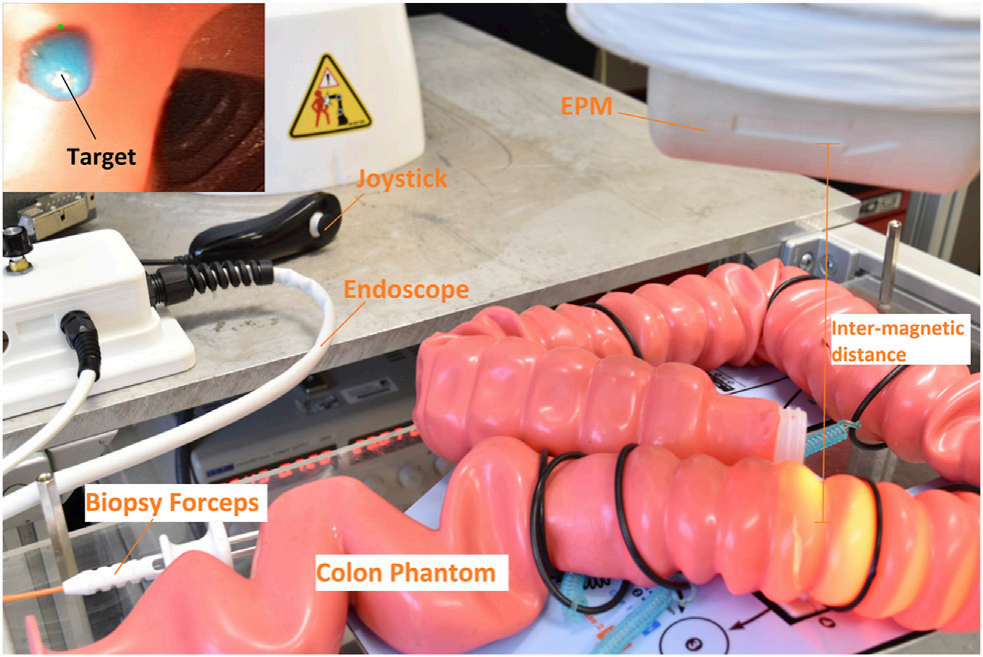
Experimental setup. The overall system is composed by an EPM, which actuates the MFE (placed inside the colon simulator), and a joystick to steer the endoscope. On the top left, the on-board camera shows a biopsy target inside a latex colonoscopy training phantom (M40, Kyoto Kagaku Co.).

The control strategy is validated on the MFE platform in a benchtop colon simulator. The platform is shown in Figure 1 and a detail of the setup is shown in Figure 5. The system is composed by a soft-tethered endoscope (the MFE) and a robotic manipulator to which an EPM is attached as the end-effector. The IPM is embedded in a 3D printed shell which comprises a LED, a camera and an instrument port. The soft-tether contains channels that provide insufflation, irrigation and a lumen dedicated to endoscopic instruments. The latter permits to insert tools such as biopsy forceps or polypectomy snares through the instrument port on the tip. The magnetic actuation is achieved by moving the EPM, attached to the flange of a serial manipulator (KUKA LBR Med R820) and, thus, imparting magnetic forces and torques on the MFE. The IPM is a cylindrical permanent magnet with an axial magnetization of 1.48 T (N52), diameter of 10.16 mm, length of 35 mm and a mass of 23 g. The EPM is a permanent magnet with a diameter and length of 101.6 mm and an axial magnetization of 1.48 T (N52). A flexible circuit, embedded in the MFE tip, is used for endoscope localization (Taddese et al., 2018). The localization algorithm estimates the MFE pose in real time (100 Hz) with a positional and rotational accuracy of 5 ± 1 mm and 6 ± 0.8° (Taddese et al., 2018).

We compared the control strategy described in Section 2 to the intelligent teleoperation control method (based on a PID controller) used in (Martin et al., 2020). This control approach was used to successfully navigate the same endoscope *in vivo* and represents the state of the art. Herein, the disturbance is proposed as the introduction of the biopsy forceps through the instrument port of the endoscope; the control is asked to actively stabilize the endoscope, counteracting any negative effects on the orientation of the tip.

We performed four biopsies on a Kyoto Kagaku M40 Colonoscope Training Simulator in *standard configuration*, Boston Scientific Single-Use Radial Jaw^TM^ 4 Biopsy Forceps were used as endoscopic tool, as shown in Figure 5. The target polyps were simulated with a blue colored polyvinyl acetate glue in different parts of the colon phantom (i.e., sigmoid, descending, transverse and ascending colon) and at different angles w.r.t. the MFE orientation. This setup aims to show that our approach can stabilize the endoscope and maintain a stable point of view of the MFE in a range of orientations that replicate the most commonly occurring cases in clinical practice. The experiments were repeated at two different inter-magnetic distances (15 and 20 cm), showing that at higher distances a more advanced controller is required to effectively stabilize the endoscope properly.

We performed 5 experiments at each inter-magnetic distance. At each experiment the user (with no prior endoscopic experience, but knowledge of the platform) was asked to perform four biopsies in four different regions of the colon. The endoscope was placed at the end of the ceacum and the user was instructed to withdraw the MFE and perform a biopsy every time a polyp was detected. Before performing the biopsy, the user was required to align the endoscope to the target by means of a joystick, using teleoperation algorithm (Martin et al., 2020). The IPM orientation at the beginning of the biopsy procedure was recorded and used as the desired target orientation in the stabilization phase.

**FIGURE 6:**
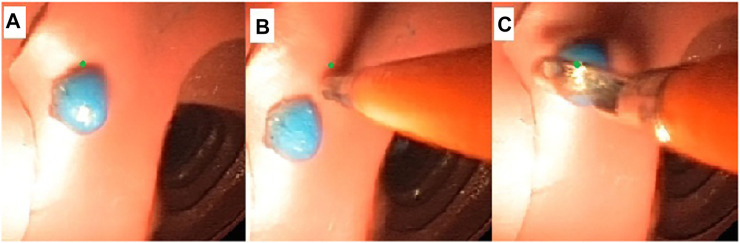
Targeted biopsy routine. **(A)** The polyp is detected by the user. The green dot is an estimated projection of the biopsy tool given an average distance of the endoscope from the target (Martin et al., 2021). **(B)** The insertion of the biopsy forceps through the working channel leads to a deviation of the tip of the endoscope to the target. **(C)** The control algorithm generates a torque on the MFE to minimize the error between the target and the tool-tip.

In Figure 6 the three main phases of the targeted biopsy routine are shown. When a polyp was detected, the user switched to the biopsy controller (i.e., LPV control) on the Graphical User Interface (GUI) and a green dot was visualized on the screen. This was an estimated projection of the biopsy tool given an average distance of the endoscope from the target tissue (this should be read as an indication of the position of the tip of the biopsy forceps when this is extruded from the tip of the endoscope, but it does not have any role in the control). While the instrument was inserted in the working channel a deviation of the tip of the endoscope from the target was induced. The control algorithm actuated the EPM, which was translated into a torque imparted on the MFE (based on the orientation error of the endoscope) that minimized the error between the target and the tool-tip. A video describing the experiments is attached. The experiments, showed in the video, are performed in the most similar possible conditions; this means same section of the colon and same position of the polyp inside the colon simulator. However, other factors such as the EPM position or the position of the tether influence the orientation of the endoscope. The video shows the PID controller is not always able to counteract the disturbance, which induces a deviation on the tip of the endoscope, resulting in fluctuation of the real IPM magnetic moment around the desired value.

**FIGURE 7:**
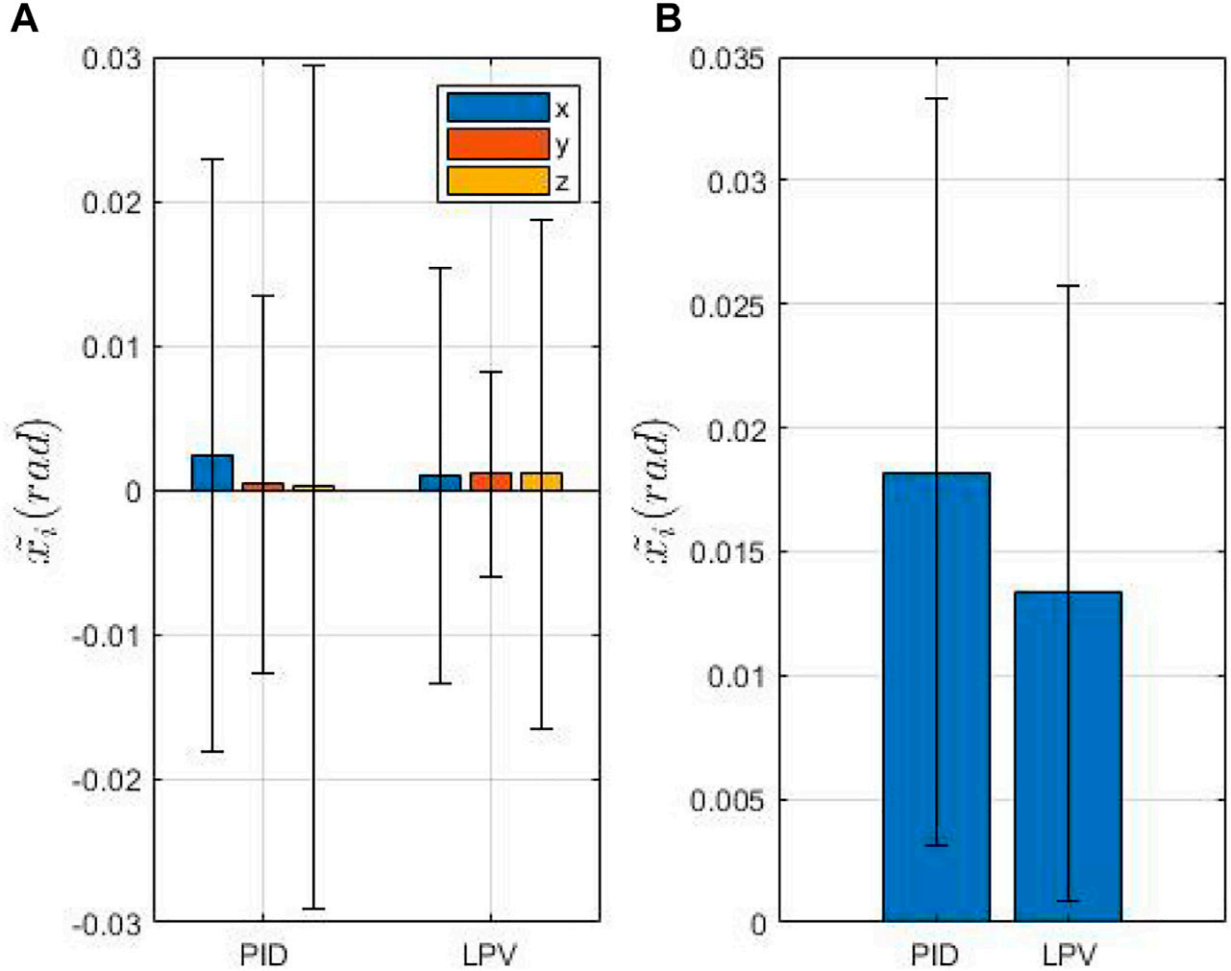
Overview of the IPM orientation error at 15 cm of inter-magnetic distance. **(A)** shows the mean and standard deviation of the orientation error. **(B)** shows the mean and the standard deviation of the euclidean norm of the mean orientation error.

**FIGURE 8:**
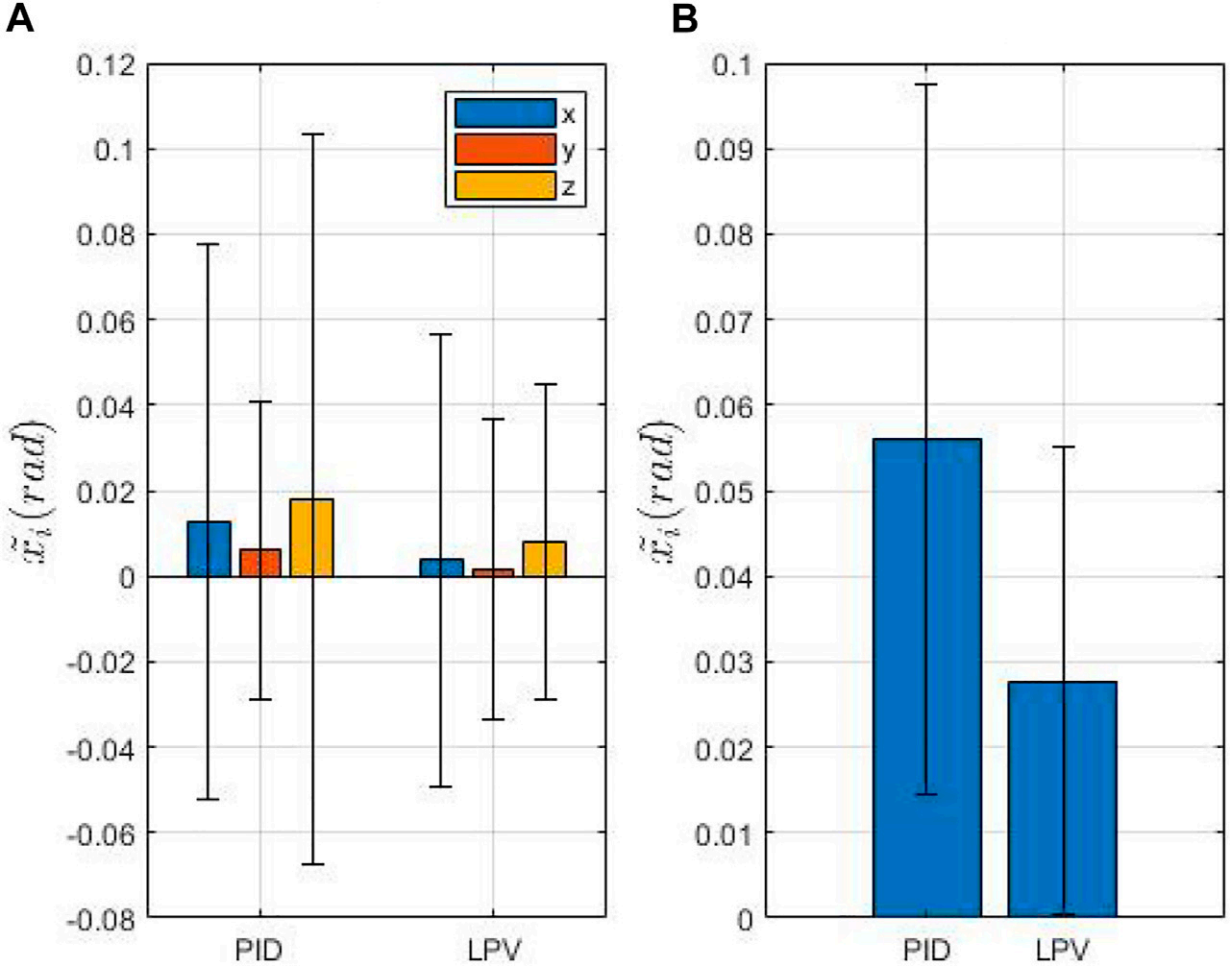
Overview of the IPM orientation error at 20 cm of inter-magnetic distance. **(A)** shows the mean and standard deviation of the orientation error. **(B)** shows the mean and the standard deviation of the euclidean norm of the mean orientation error.

Figure 7A and Figure 8A show the mean error (defined as $1/n\sum_{i=1}^{n}{\tilde{x}}_{i}$ , where ${\tilde{x}}_{i}$ is the MFE orientation error at each cycle and *n* the number of cycles) along each axis, Figures 7B, 8B the Euclidean norm of the error. In both figures, we compare the LPV approach with the algorithm discussed in (Martin et al., 2020). In Figure 7, the EPM is placed at the minimum height (0.15 m), while in Figure 8 the inter-magnetic distance is set to the maximum (0.2 m). These values have been chosen as extremes of a safe window in which a possible collision with the patient is avoided and simultaneously the transmittable torques are still significant. As expected, the inter-magnetic distance is correlated with the error in both cases. At the maximum distance, the steerability of the MFE decreases, especially when the biopsy tool is inserted inside the working channel. The tool increases the stiffness of the tether and, thus, the magnetic torque occasionally ineffectively orientates the MFE. However, although the mean error increases with the relative distance, our approach is able to effectively reduce the error compared to the PID controller. In particular, we observe a reduction on the norm of 18.1 and 45.8*%* at the lowest and the highest inter-magnetic distance, respectively. The difference is larger at higher inter-magnetic distance (Figure 8), showing that control adaptation with respect to distance can significantly improve performances. The z-test, applied to the experimental data, confirms the statistical significance of the results with a *p*-value <0.05 .

Table 1 reports the absolute mean orientation error and the relative standard deviation on each axis, with both approaches. We notice that, at the lowest inter-magnetic distance (Figure 7A), the absolute mean error is low and comparable with both approaches, while the standard deviation is substantially higher with the PID controller. The LPV controller presents a reduction of the relative percentage of the error on the x axis equal to 57.79*%*, but a higher relative percentage of the error on the *y* and *z* axis (175 and 250*%*). However, since the absolute mean values of the error, as reported in Table 1, are significantly low for both controllers, while the standard deviation is higher for the PID controller, the two approaches can be considered comparable.

**TABLE 1:** Mean and standard deviation of the orientation error at different inter-magnetic distances.

*ρ* (m)	Axis	PID (rad)	LPV (rad)
0.15	x	0.002 ± 0.021	0.001 ± 0.0144
	y	0.0004 ± 0.013	0.001 ± 0.0071
	z	0.0002 ± 0.0292	0.0011 ± 0.0176
0.20	x	0.0129 ± 0.0650	0.0037 ± 0.0529
	y	0.0061 ± 0.0347	0.0016 ± 0.0351
	z	0.0178 ± 0.0853	0.0081 ± 0.0368

On-the-other-hand, taking into account the highest inter-magnetic distance (Figure 8A), we can notice that the mean error and the standard deviation are significantly reduced with the LPV controller compared to the PID, as reported in Table 1. In fact, at the highest inter-magnetic distance, our method obtains a reduction of 70.8*, 74.2* and 54.5*%* on the *x*, ,*y* and *z* axis, respectively, compared to the PID controller.

**FIGURE 9:**
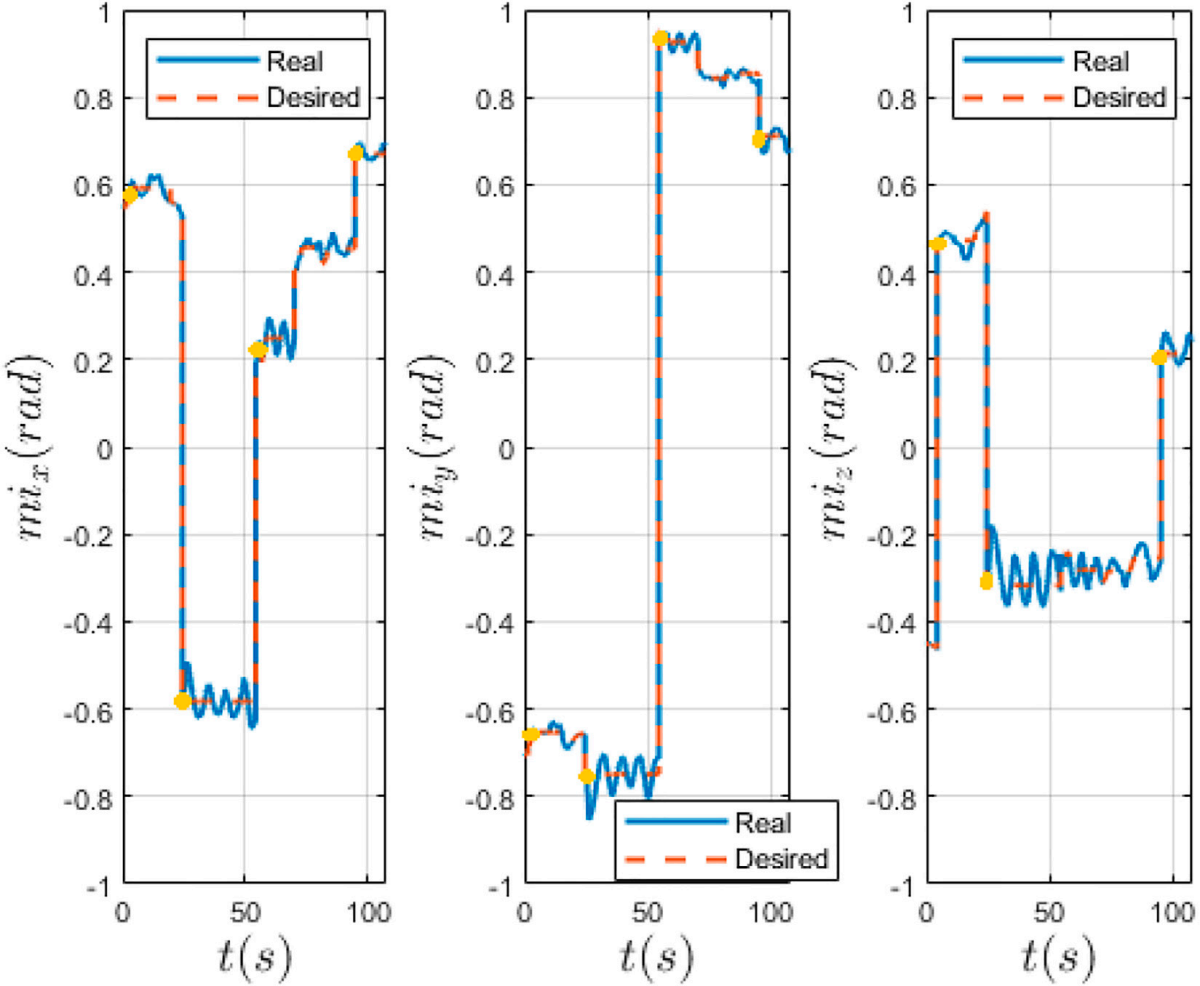
Real and desired IPM magnetic moment computed with PID controller. Desired (red) and real (blue) magnetic moment of the IPM in world frame. The yellow dots indicate the moment when the biopsy forceps is being introduced in the instrument port.

**FIGURE 10:**
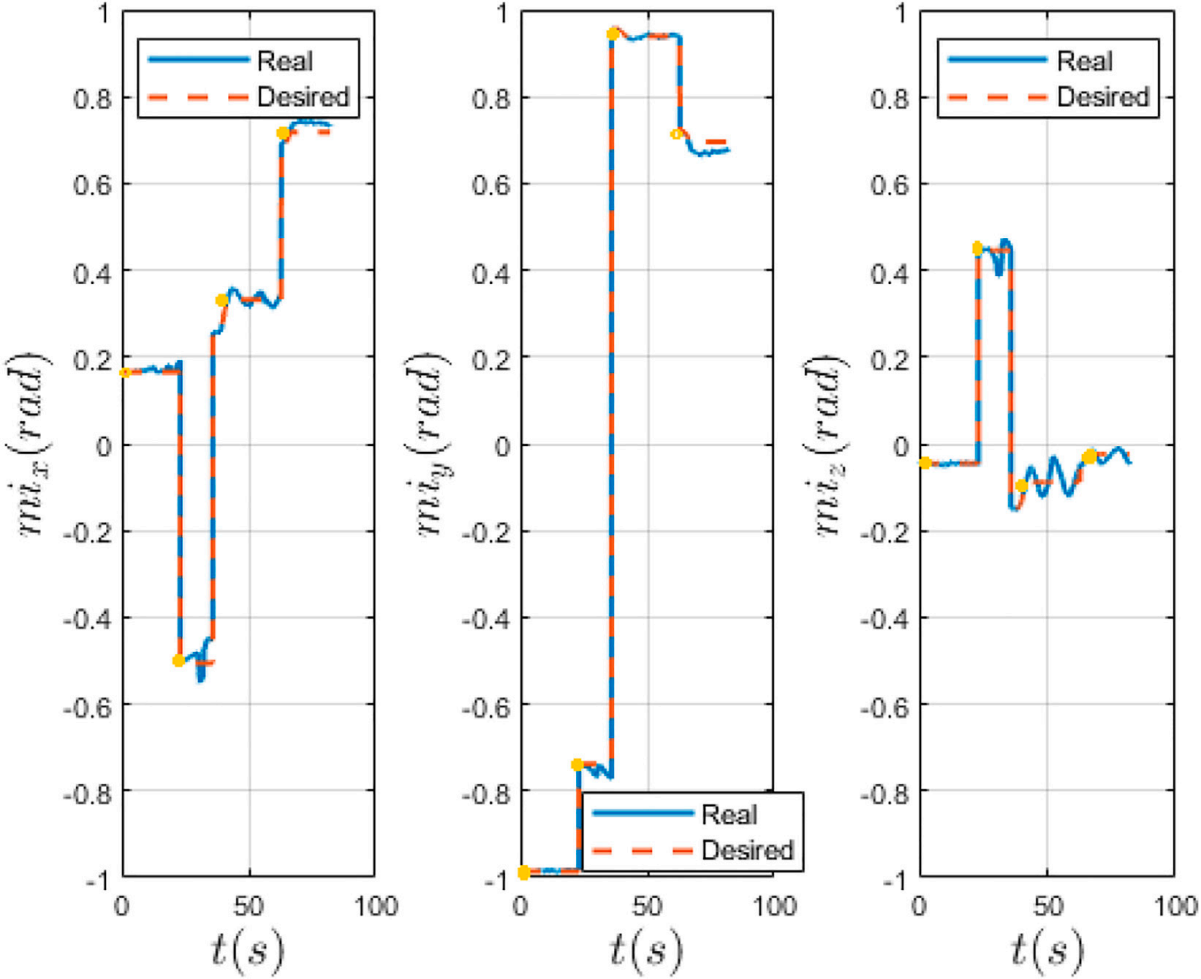
Real and desired IPM magnetic moment computed with LPV controller. Desired (red) and real (blue) magnetic moment of the IPM in world frame. The yellow dots indicate the moment when the biopsy forceps is being introduced in the instrument port.

In Figures 9, 10 we compare the PID and LPV approaches by evaluating the measured and desired magnetic moment of the MFE, defined by the user by means of a joystick before switching to the biopsy controller on the GUI. The experiment that had the lowest mean error, at the highest inter-magnetic distance, for both approaches is shown. Both approaches are effective, however, the PID controller presents more oscillations around the desired value, generated by external disturbances (i.e. insertion of the biopsy forceps inside the instrument port) and less effectively damped by the PID. Our approach presents lower oscillations, showing that our method is able to stabilize the system in the presence of external disturbances. In Table 2, we quantify the fluctuations (i.e., standard deviation) of the measured magnetic moment around the desired value, with both approaches and the percentage reduction of the LPV approach w.r.t. the PID controller, with regard to the experiments reported in Figures 9, 10.

**TABLE 2:** Quantitative analysis of the magnetic moment fluctuations.

Axis	PID (rad)	LPV (rad)	Reduction (%)
x	0.0738	0.0302	59.08
y	0.0434	0.0255	41.32
z	0.0740	0.0622	16.0

**FIGURE 11:**
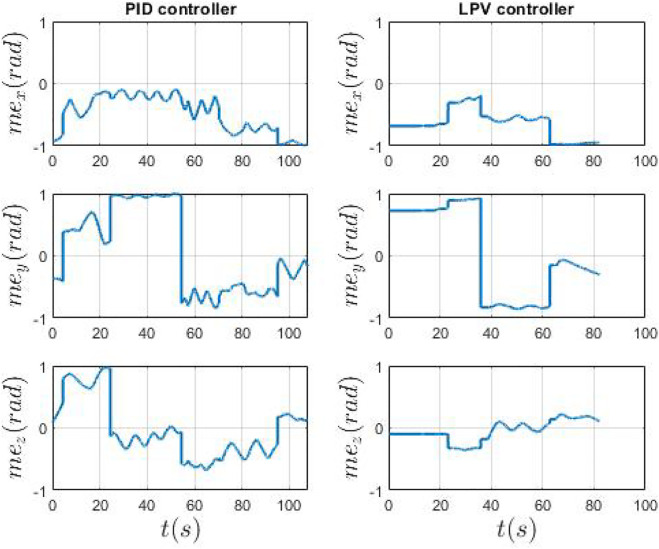
Real EPM magnetic moment in world frame. On the left the PID control and on the right the LPV controller. The EPM magnetic moment is computed by the controller, at each cycle, given the IPM magnetic moment error.

Figure 11 shows the variation of EPM magnetic moment with both controllers. It is worth noting that our approach significantly reduces the movements of the EPM showing that a more effective control action is computed with the LPV controller. Moreover, limiting the movement of the EPM permits to reduce the risk of the robot contact with the patient, avoiding potentially dangerous situations. The LPV control achieves a maximum reduction of the EPM oscillations of 58.1*%*, computed on the mean value of the magnetic moment, compared to the PID controller.

## 4 Discussion

This paper discusses a model-based LPV control approach with the aim of stabilizing a magnetically manipulated endoscope during interventional procedures such as biopsy, polyp removal and clip placement. We show the application of the proposed technique on the MFE, used to perform targeted biopsies. We prove the proposed method is capable of stabilizing the endoscope in the presence of external disturbances (i.e., insertion of the tool in the instrument port) and provides enhanced performances with respect to the literature. This method can aid interventional tasks by enhancing the accuracy of the biopsy procedure. Moreover, the active stabilization of the endoscope allows a single user to perform each task autonomously and to reduce the number of people in the endoscopy room (Onaizah et al., 2021).

The control strategy is based on the LPV control that facilitates stabilization of the endoscope in the working environment. The novelty of this approach is the fact that linearizing the non-linear system in different equilibria (i.e., inter-magnetic distance between the two magnets) permits to cope with the different status of the non-linear system. In particular, the magnetic force and torque the EPM imparts on the IPM are strictly related to the relative distance between the two magnets: at higher inter-magnetic distances, due to various factors (i.e. different anatomies of the patients, position of the patient on the bed), the magnetic torque drops significantly. Therefore, a suitable robust controller able to cope with variations in the inter-magnetic distance is crucial. To our knowledge, this is the first example of LPV control synthesis applied to a magnetic endoscope. Embedding this method in a clinical scenario could improve the clinical performance and ease-of-use of interventional tasks such as biopsy, polyp removal and clip placement.

To prove the strength of our approach, we performed 5 trials on a colon phantom. Four biopsies in different sections of the colon and at different orientation were taken using a biopsy forceps. The results show both approaches are effective, but the LPV approach was able to obtain a lower orientation error with respect to the PID controller. In particular, it is worth noting that at the maximum height, the oscillations around the desired magnetic moment direction are reduced by 45.8*%* with our method. Using an optimized and model-based controller, which takes into account different system equilibria, has the advantage of robustness, withstanding parameter variation still maintaining stability and performance goals. However, the main limitation of this work, as shown by Figures 9, 10 and the video attached to the paper, is the fact that a direct comparison of the experiments is not straightforward. In fact, the user inputs may vary and, thus, the experiments are not very repeatable. Nonetheless, the same conditions for each experiment were tried to replicate.

In the future, we will integrate our method with an Artificial Intelligence (AI) (Mitsala et al., 2021) or a semi-autonomous routine (Martin et al., 2021) to target biopsies. Herein, the authors were able to track a target tissue, predict the projection of the tool channel outside the tip of the endoscope using a stereo-vision approach and align the magnetic endoscope to the polyps; however, no additional disturbances were taken into account. Combining (Martin et al., 2021) and our approach (i.e. the tracking of a tissue target and the active stabilization of the endoscope) we could achieve a completely autonomous procedure and reduce the personnel needed in the room. Although the discussion with clinical operators highlighted that the disturbances introduced by the instrument are the most disruptive, further works should investigate and adapt our approach in the context of additional disturbances such as patient breathing and peristalsis, that might require tracking of the tissues and target.

## Data Availability

The raw data supporting the conclusion of this article will be made available by the authors, without undue reservation.
